# WHO’S AFRAID OF LIVING TO 100? ATTITUDES TOWARD A CENTENARIAN LIFE ACROSS GENERATIONS

**DOI:** 10.1093/geroni/igae098.3282

**Published:** 2024-12-31

**Authors:** Adam Felts, Lauren Cerino, Taylor Patskanick, Lisa D’Ambrosio, Joseph Coughlin

**Affiliations:** Massachusetts Institute of Technology, Cambridge, Massachusetts, United States; Massachusetts Institute of Technology, Cambridge, Massachusetts, United States; Massachusetts Institute of Technology, Cambridge, Massachusetts, United States; MIT AgeLab, Cambridge, Massachusetts, United States; MIT AgeLab, Cambridge, Massachusetts, United States

## Abstract

The U.S. centenarian population is expected to increase fourfold over the next three decades, according to Pew Research Center (2024). Amid this increasing likelihood of living to 100, and given the general mystique that a century-long life possesses, how do people think and feel about the possibility of a 100-year life? We explore results from a survey that asked respondents a battery of questions related to their attitudes toward a 100-year lifespan, including their interest in living a 100-year life, their emotions, their concerns, and their perceived level of preparedness. Further, we examine the sources of advice that respondents trusted to guide them through a 100-year life. Finally, we review archetypes respondents chose that reflect the kind of person they would like to embody at age 100, to begin to explore questions (and how to ask questions) about how people conceptualize the “the good life” in later old age. Reporting and discussion of survey results (N=1,184) will be paired with qualitative findings from focus groups (N=69) on the same topic. In the discussion of these results, we will focus on whether people hope for or fear a 100-year life, what their aspirations would be in old age given a lifespan of such great length, and how they would prepare for it. Particular attention will be paid to generational or life course differences in responses, especially in the sources of advice that people prefer, which suggest profound generational shifts in people’s preferred sources of expertise.

